# Identification of prognostic significance of *BIRC5* in breast cancer using integrative bioinformatics analysis

**DOI:** 10.1042/BSR20193678

**Published:** 2020-02-18

**Authors:** Jian-bo Dai, Bei Zhu, Wei-jia Lin, Hai-yan Gao, Hong Dai, Lin Zheng, Wei-hai Shi, Wei-xian Chen

**Affiliations:** 1 Department of Breast Surgery, The Affiliated Changzhou No.2 People’s Hospital of Nanjing Medical University, Changzhou 213000, Jiangsu Province, China; 2 Post-doctoral Working Station, The Affiliated Changzhou No.2 People’s Hospital of Nanjing Medical University, Changzhou 213000, Jiangsu Province, China

**Keywords:** Biomarker, BIRC5, Breast cancer, Prognosis

## Abstract

**Aims:** Baculoviral inhibitor of apoptosis repeat containing 5 (*BIRC5*) plays vital roles in carcinogenesis by influencing cell division and proliferation and by inhibiting apoptosis. However, the prognostic significance of *BIRC5* remains unclear in breast cancer.

**Methods:** *BIRC5* expression and methylation status were evaluated using the Oncomine and The Cancer Genome Atlas (TCGA) databases. The relevance between *BIRC5* and different clinicopathological features as well as survival information was analyzed using the bc-GenExMiner database and Kaplan–Meier Plotter. *BIRC5*–drug interaction network was obtained using the Comparative Toxicogenomics Database.

**Results:** Based on the results from databases and own hospital data, *BIRC5* was higher expressed in different breast cancer subtypes compared with the matched normal individuals. Hormone receptors were negatively correlated with *BIRC5* expression, whereas the Scarff–Bloom–Richardson (SBR) grade, Nottingham Prognostic Index (NPI), human epidermal growth factor receptor-2 (HER-2) status, basal-like status, and triple-negative status were positively related to *BIRC5* level in breast cancer samples with respect to normal tissues. High *BIRC5* expression was responsible for shorter relapse-free survival, worse overall survival, reduced distant metastasis free survival, and increased risk of metastatic relapse event. *BIRC5*–drug interaction network indicated that several common drugs could modulate *BIRC5* expression. Furthermore, a positive correlation between *BIRC5* and*cell-division cycle protein 20* (*CDC20*) gene was confirmed.

**Conclusion:** *BIRC5* may be adopted as a promising predictive marker and potential therapeutic target in breast cancer. Further large-scale studies are needed to more precisely confirm the value of *BIRC5* in treatment of breast cancer.

## Introduction

Breast cancer is now the most common malignancy among women worldwide [1,2]. Although precise surgery and adjuvant systemic treatments including chemotherapeutic agents, radiotherapy, hormone therapy, and molecular targeting drugs greatly improve the overall outcome, the prognosis of breast cancer remains poor. The limitation of clinical, pathological, and molecular features in individualized tumor therapy urgently requires a novel approach to predict outcome and treatment response. Therefore, finding some more available and effective markers as surrogates of these features is of crucial importance [3].

As a mitotic spindle checkpoint gene, baculoviral inhibitor of apoptosis repeat containing 5 (*BIRC5*, also known as survivin) has been shown to play vital roles in carcinogenesis by influencing cell division and proliferation and by inhibiting apoptosis [4]. Since *BIRC5* is frequently overexpressed in a majority of malignancies [5–7], treatment that targets *BIRC5* has been increasingly noticed as a novel strategy for various malignant tumors. For example, inactivation of nuclear export signal for *BIRC5* may increase therapy response in head and neck cancer patients [8]. Both molecular suppression by gene editing approach and pharmacological inhibition by *BIRC5* antagonist could reduce the growth, migration, and invasiveness of ovarian cancer cells [7]. In terms of breast cancer, *BIRC5* was highly expressed in triple-negative subtype and *BIRC5* repression was able to decrease the proliferation of breast cancer cells, implying that *BIRC5* acts like a tumor driver [9]. Moreover, it was demonstrated that *BIRC5* was a pejorative marker in stage II/III breast cancer with no response to neoadjuvant chemotherapy [10]. Besides, *BIRC5* expression has been found to confer resistance to chemotherapy and radiation. Targeting *BIRC5* in experimental models improves survival [11]. Together, these findings indicate that *BIRC5* may not only function as an oncogene, but also as a promising predictive biomarker and potential therapeutic target in cancer [12].

Therefore, the present work was carried out to validate the expression pattern, potential function, prognostic value, and drug interaction network of *BIRC5* in breast cancer by performing bioinformatics analysis of several large online databases.

## Materials and methods

### Breast tissue samples

A total of 18 pairs of breast tissue samples were obtained from the Affiliated Changzhou No.2 People’s Hospital of Nanjing Medical University between 2014 and 2016. The collection and use of samples was conducted in accordance with the Declaration of Helsinki and approved by the ethics committee of Changzhou No.2 People’s Hospital [13]. Informed written consent was received from all patients.

### Oncomine

The Oncomine (http://www.oncomine.org), a database containing publicly available microarray data on multiple tumors, was searched to check the level of *BIRC5* in breast cancer and normal tissues with the options as follows: fold change ≥2, *P*-value ≤1E-4, and the top 10% gene ranking [14]. *BIRC5* co-expression profile was shown in the heatmap.

### Ualcan

The Ualcan (http://ualcan.path.uab.edu/), a popular web source for in-depth analysis of The Cancer Genome Atlas (TCGA) data, was used to validate the relative level of *BIRC5* across tumor and normal samples, as well as in tumor subgroups according to different stages [15]. Besides, the promoter methylation level of *BIRC5* in breast cancer and normal tissues was obtained by using Ualcan.

### bc-GenExMiner

The Breast Cancer Gene-Expression Miner v4.1 (bc-GenExMiner v4.1, http://bcgenex.centregauducheau.fr/BC-GEM), an open access database of published annotated genomics data, was utilized to analyze the relevance between *BIRC5* and specific clinicopathological features of breast cancer [16,17]. Association between *BIRC5* and metastatic relapse event was assessed using the prognostic module, and correlation of *BIRC5* and co-expressed *cell-division cycle protein 20* (*CDC20*) was evaluated using the correlation module.

### UCSC Xena

The UCSC Xena browser (http://xena.ucsc.edu/) was used to analyze the TCGA Breast Cancer data using the level 3 data. The heatmap and correlation between *BIRC5* and *CDC20* were then constructed.

### Kaplan–Meier Plotter

The Kaplan–Meier Plotter (http://kmplot.com/analysis/), a web tool capable to check the effect of 54675 genes on survival using 5143 clinical breast cancer samples, was applied to show the prognostic value of *BIRC5* in relapse-free survival, overall survival, and distant metastasis-free survival [18]. The hazard ratio (HR) with 95% confidence interval (CI), and log-rank *P*-value were calculated automatically.

### Comparative Toxicogenomics Database

The Comparative Toxicogenomics Database (http://ctdbase.org/) was employed to construct *BIRC5*–drug interaction network [19]. Concretely, *BIRC5* was checked in the database for potential chemical drugs that could decrease/increase the mRNA or protein expression of *BIRC5*. Drugs were picked based on their clinical applications in breast cancer treatment. *BIRC5*–drug interaction network was then generated using the Cytoscape (http://cytoscape.org/) [20,21]. *BIRC5* gene was also queried in the database and the results were filtered by selecting *BIRC5*-related pathways.

### RNA isolation and real-time PCR and Western blot

Total RNA was extracted using the TRIzol reagent (TaKaRa, Japan), and 1000 ng RNA was reverse transcribed into cDNA using the SYBR PrimeScript RT-PCR kit (Takara Bio Inc., Japan) on an iCycler iQ system (Bio-Rad, U.S.A.). Real-time PCR was performed using the same kit on a Light Cycler 480 (Roche, Australia). The primer sequence of *BIRC5* and endogenous control *β-actin* was as follows: forward, 5′‐ATTCGTCCGGTTGCGCTTTCC‐3′; reverse, 5′‐CACGGCGCACTTTCTTCGCAG‐3′; and forward, 5′-GCTGTGCT ATCCCTGTACGC-3′; reverse, 5′-TGCCTCAGGGCAGCGGAACC-3′, respectively. All reactions, including the negative controls, were performed in triplicate.

Tissue proteins were extracted, electrophoresed through SDS/PAGE gel, and transferred to PVDF membranes for Western blot. *BIRC5* protein level was quantified using antibody against *BIRC5* (1:1000, Santa Cruz, U.S.A.). β-actin (Sigma, Germany) was used for normalization. Bound proteins were visualized by using the ECL Plus Kit (Millipore, U.S.A.) with Image Lab Software (Bio-Rad, U.S.A.).

### Statistical analysis

According to protocols of the above tools, mRNA levels of *BIRC5* in breast cancer and normal tissues in each individual dataset were analyzed using the Student’s *t* test. Kaplan–Meier survival analysis was performed to compare patient survival based on *BIRC5* expression by log-rank test. Global significant difference between groups of clinical parameters was assessed by Welch’s test, along with Dunnett–Tukey–Kramer’s.

## Results

### *BIRC5* expression and methylation status in breast cancer patients

**Figure 1:**
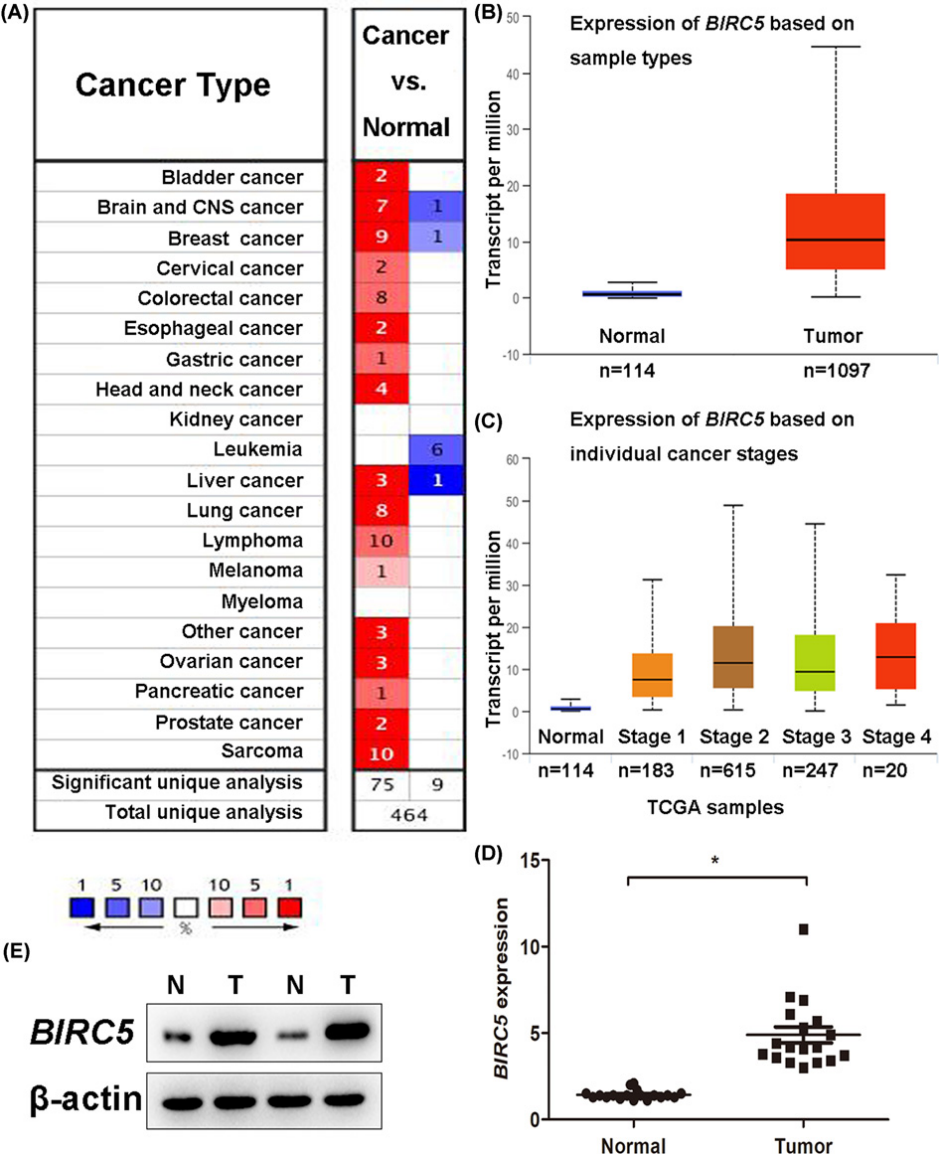
*BIRC5* expression in breast cancer patients (**A**) Expression of *BIRC5* in 20 types of cancer and the matched normal individuals using the Oncomine database. Red and blue indicate the numbers of datasets with statistically significant (*P*<0.05) higher expressed and lower expressed *BIRC5* gene. (**B**) Higher mRNA *BIRC5* was expressed in breast cancer tissues than in normal tissues (*P*<0.05) using Ualcan online tool. (**C**) Patients with a more advanced stage of breast cancer tended to express higher levels of *BIRC5* using Ualcan online tool. (**D**) Expression of *BIRC5* in 18 pairs of breast cancer tissues and adjacent normal breast tissues obtained from our own hospital. (**E**) *BIRC5* protein expression in two pairs of breast cancer tissues and adjacent normal breast tissues (*, P<0.05).

**Figure 2:**
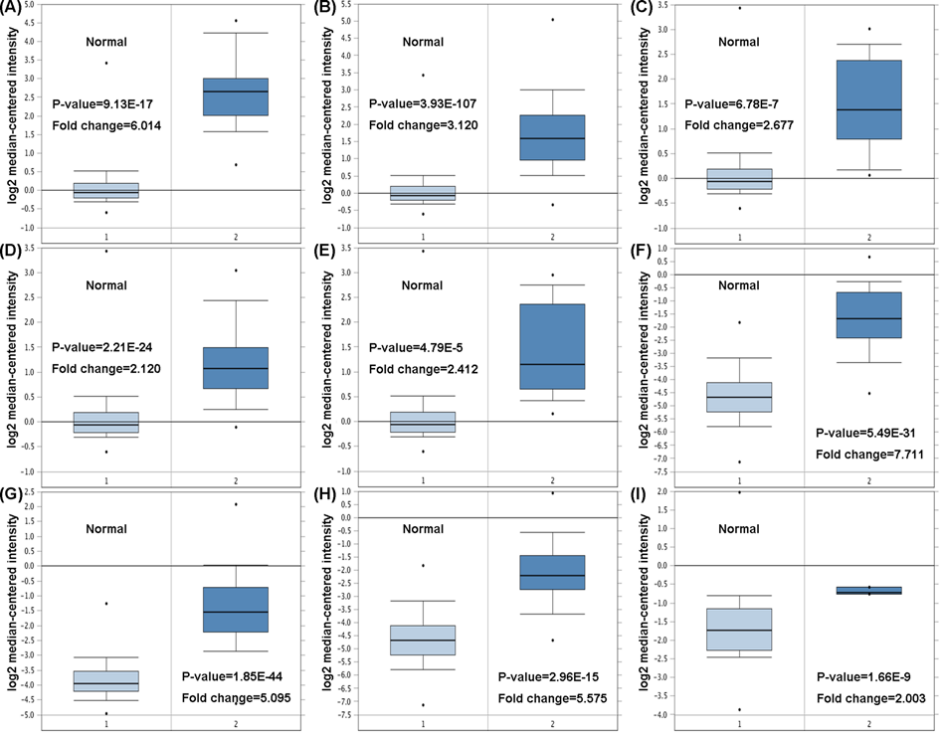
Box plot comparing *BIRC5* expression in normal tissues and breast cancer patients obtained from the Oncomine database Analysis is shown for (**A**) medullary breast carcinoma, (**B**) invasive ductal breast carcinoma, (**C**) invasive breast carcinoma, (**D**) invasive ductal and invasive lobular breast carcinoma, (**E**) breast carcinoma, (**F**) invasive breast carcinoma, (**G**) invasive ductal breast carcinoma, (**H**) invasive lobular breast carcinoma, and (**I**) intraductal cribriform breast adenocarcinoma (*, P<0.05).

**Figure 3:**
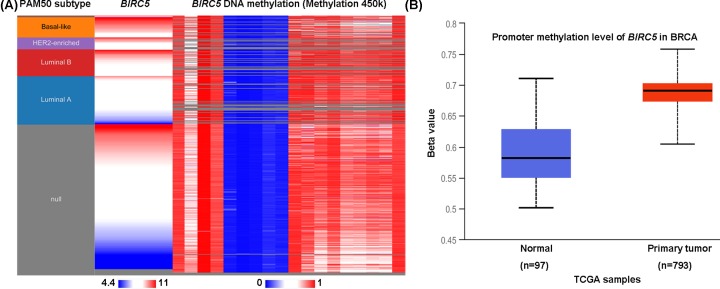
*BIRC5* methylation status in breast cancer patients (**A**) Heatmap of *BIRC5* expression and DNA methylation status using the TCGA data analyzed by the UCSC Xena browser. (**B**) Higher promoter methylation level of *BIRC5* was observed in breast cancer tissues, with respect to normal tissues.

We first measured the *BIRC5* levels in 20 cancer types. Oncomine database revealed that *BIRC5* was significantly higher expressed in breast cancer patients compared with the matched normal individuals (Figure 1A). TCGA data analyzed by the Ualcan online tool confirmed that higher *BIRC5* was expressed in breast cancer tissues than in normal tissues (Figure 1B, *P*<0.05). Moreover, patients with a more advanced stage of breast cancer tended to express higher levels of *BIRC5* (Figure 1C.) Consistent with the results from databases, we also compared the *BIRC5* expression in breast cancer tissues and adjacent normal tissues in our hospital and found that *BIRC5* was elevated in breast cancer tissues both in mRNA level (Figure 1D) and protein level (Figure 1E). Specifically, increased level of *BIRC5* was observed in medullary breast carcinoma, invasive ductal breast carcinoma, invasive breast carcinoma, invasive ductal and invasive lobular breast carcinoma, breast carcinoma, invasive lobular breast carcinoma, and intraductal cribriform breast adenocarcinoma with respect to normal tissues (Figure 2A–I). Heatmap and DNA methylation status indicated that *BIRC5* expression might be negatively associated with DNA methylation in breast cancer (Figure 3A). Besides, higher promoter methylation level of *BIRC5* was observed in breast cancer tissues, with respect to normal tissues (Figure 3B).

### *BIRC5* expression and clinicopathological features in breast cancer patients

**Figure 4:**
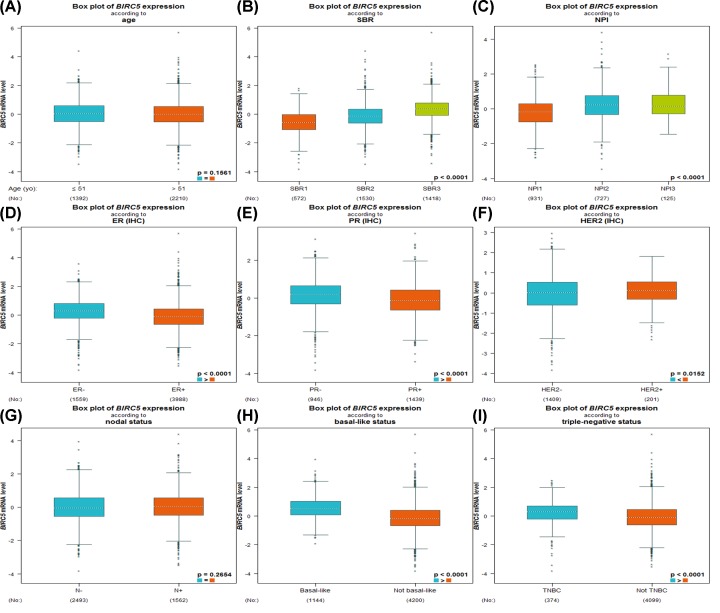
Box plot evaluating the relevance of *BIRC5* expression and different clinicopathological features using the bc-GenExMiner web-based tool Analysis is shown for (**A**) age, (**B**) SBR, (**C**) NPI, (**D**) ER, (**E**) PR, (**F**) HER-2, (**G**) nodal status, (**H**) basal-like status, and (**I**) triple-negative status. Abbreviations: ER, estrogen receptor; PR, progesterone receptor (*, P<0.05).

We checked the relevance of *BIRC5* expression and different clinicopathological features by using the bc-GenExMiner web-based tool. No significant difference could be found between ≤51- and >51-year groups (Figure 4A and Table 1). Breast cancer patients with more advanced Scarff–Bloom–Richardson (SBR) grade and Nottingham Prognostic Index (NPI) showed elevated *BIRC5* gene [22,23] (Figure 4B,C). Hormone receptor status was found to correlate negatively with *BIRC5* (Figure 4D,E and Table 1); whereas human epidermal growth factor receptor-2 (HER-2) status was positively associated with *BIRC5* level (Figure 4F and Table 1). In terms of nodal status, no significant difference was found between positive group and negative group (Figure 4G and Table 1). Our results also demonstrated strongly higher *BIRC5* expression in basal-like and triple-negative breast cancer patients with respect to non-basal-like and non-triple-negative patients (Figure 4H,I and Table 1).

**Table 1:** Relevance of *BIRC5* expression and different clinicopathological features using the bc-GenExMiner database

Variables	Number of patients	*BIRC5* mRNA	*P*-value
**Age (years)**			0.1561
≤51	1392	-	
>51	2210	-	
**ER**			<0.0001
Negative	1559	Increased	
Positive	3988	-	
**PR**			<0.0001
Negative	946	Increased	
Positive	1439	-	
**HER-2**			0.0152
Negative	1409	-	
Positive	201	Increased	
**Nodal status**			0.2654
Negative	2493	-	
Positive	1562	-	
**Basal-like status**			<0.0001
Non-basal-like	4200	-	
Basal-like	1144	Increased	
**Triple-negative status**			<0.0001
Non-triple-negative	4099	-	
Triple-negative	374	Increased	

Abbreviation: ER, estrogen receptor; PR, progesterone receptor.

### *BIRC5* expression and prognosis in breast cancer patients

**Figure 5:**
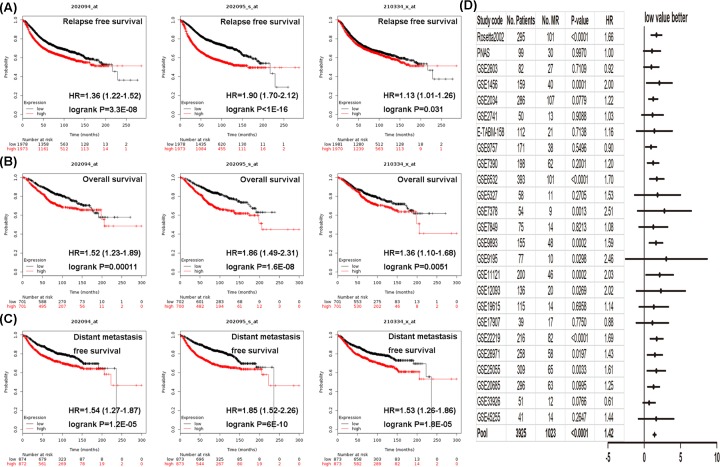
Survival curves derived from the Kaplan–Meier Plotter and forest plot generated from the bc-GenExMiner database evaluating the prognostic significance of *BIRC5* Analysis is shown for (**A**) relapse-free survival, (**B**) overall survival, (**C**) distant metastasis-free survival, and (**D**) metastatic relapse event.

We then investigated the prognostic value of *BIRC5* gene using the Kaplan–Meier Plotter. While breast cancer patients with lower level of *BIRC5* showed longer relapse-free survival and better overall survival, patients with increased *BIRC5* expression displayed shorter distant metastasis-free survival (Figure 5A–C). By mining previously available data using the bc-GenExMiner, we analyzed the association between *BIRC5* and metastatic relapse-free survival. High level of *BIRC5* was significantly associated with elevated risk of metastatic relapse event (HR = 1.42, 95% CI: 1.33–1.52, *P*<0.0001), as suggested by the forest plot (Figure 5D).

### *BIRC5*–drug interaction network

**Figure 6:**
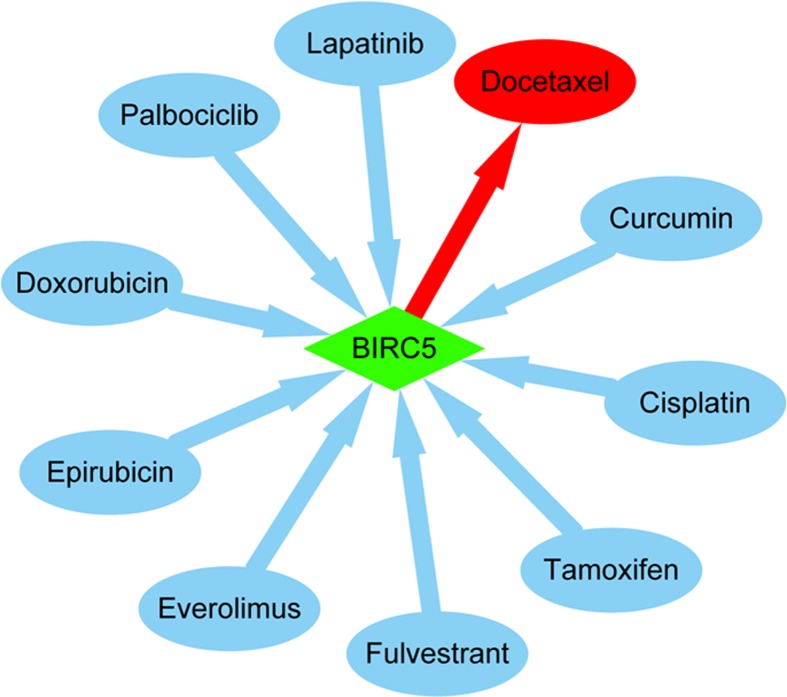
*BIRC5*–drug interaction network obtained from the Comparative Toxicogenomics Database The network shows that several common drugs could modulate the mRNA or protein expression of *BIRC5*. For example, tamoxifen could decrease *BIRC5* expression (blue), while docetaxel could increase *BIRC5* level (red).

Given that high level of *BIRC5* conferred shorter survival in breast cancer patients, we then sought to investigate whether *BIRC5* and potential chemical drugs could modulate each other using the Comparative Toxicogenomics Database. *BIRC5*–drug interaction network indicated that a number of commonly used drugs could modulate the mRNA or protein expression of *BIRC5*. Specifically, chemotherapy agents including cisplatin, epirubicin, and doxorubicin, endocrine drugs such as tamoxifen and fulvestrant, and molecular targeting drugs lapatinib and palbociclib could decrease *BIRC5* level; whereas docetaxel was able to increase *BIRC5* expression (Figure 6). By using the Comparative Toxicogenomics Database, several important *BIRC5*-related signaling pathways were also obtained including apoptosis, cell cycle, immune system, hippo signaling pathway, platinum drug resistance, pathways in cancer, and TP53 regulation (Table 2).

**Table 2:** Analysis of *BIRC5*-involved pathway terms

Pathway ID	Pathway term
KEGG: hsa04210	Apoptosis
REACT: R-HSA-1640170	Cell cycle
REACT: R-HSA-69278	Cell cycle, mitotic
REACT: R-HSA-1280215	Cytokine signaling in immune system
REACT: R-HSA-74160	Gene expression
REACT: R-HSA-212436	Generic transcription pathway
KEGG: hsa04390	Hippo signaling pathway
REACT: R-HSA-168256	Immune system
REACT: R-HSA-6785807	Interleukin-4 and 13 signaling
REACT: R-HSA-392499	Metabolism of proteins
KEGG: hsa05200	Pathways in cancer
KEGG: hsa01524	Platinum drug resistance
REACT: R-HSA-597592	Post-translational protein modification
REACT: R-HSA-449147	Signaling by interleukins
REACT: R-HSA-194315	Signaling by Rho GTPases
REACT: R-HSA-162582	Signaling transduction
REACT: R-HSA-5633008	TP53 regulates transcription of cell death genes
REACT: R-HSA-3700989	Transcriptional regulation by TP53
REACT: R-HSA-6803205	TP53 regulates transcription of several additional cell death genes whose specific roles in p53-dependent apoptosis remain uncertain

### Co-expression of *BIRC5* gene

**Figure 7:**
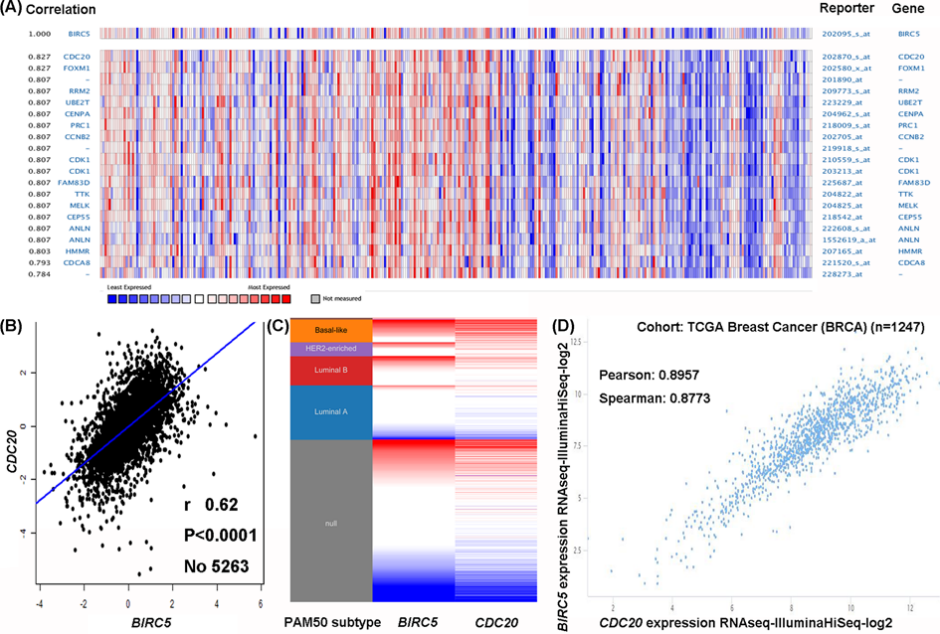
Co-expression of *BIRC5* gene (**A**) Co-expression profile of *BIRC5* analyzed using the Oncomine database. (**B**) Correlation between *BIRC5* and *CDC20* in breast cancer analyzed using the bc-GenExMiner software. (**C**) Heatmap of *BIRC5* and *CDC20* expression across PAM50 breast cancer subtypes using the TCGA data analyzed by the UCSC Xena browser. (**D**) Correlation between *BIRC5* and *CDC20* expression using the TCGA data analyzed by the UCSC Xena browser.

We finally investigated the co-expression of *BIRC5* gene using the Oncomine. *BIRC5* co-expression profile consists of a large cluster of 19574 genes from 336 breast cancer samples (Figure 7A). The most highly correlated gene was *CDC20*. *CDC20* is an important regulatory cell cycle protein that activates the anaphase-promoting complex during mitosis, resulting in chromatid separation, and entrance of cell cycle into anaphase [24]. Data mining using the bc-GenExMiner revealed that *BIRC5* was positively correlated with *CDC20* (Figure 7B). Such positive relationship was verified by analyzing TCGA database using the UCSC Xena (Figure 7C,D).

## Discussion

Based on the high activation of *BIRC5* during tumorigenesis in various cancer types, treatment that targets *BIRC5* has been increasingly noticed as a promising therapeutic strategy [11]. However, the detailed expression pattern, potential function, prognostic value, and drug interaction network of *BIRC5* remain largely unclear in breast cancer.

First, our analysis using the Oncomine and TCGA databases revealed that *BIRC5* gene was higher expressed in breast cancer patients with respect to normal individuals. Moreover, Oncomine showed that *BIRC5* was significantly elevated in medullary breast carcinoma, invasive ductal breast carcinoma, invasive breast carcinoma, invasive ductal and invasive lobular breast carcinoma, breast carcinoma, invasive lobular breast carcinoma, and intraductal cribriform breast adenocarcinoma, compared with the corresponding normal tissues.

Second, we investigated the mechanisms of *BIRC5* dysregulation in breast cancer. After checking the DNA methylation status in TCGA, we found that *BIRC5* expression was negatively related to DNA methylation and higher promoter methylation level of *BIRC5* was expressed in breast cancer tissues. These observations indicated that DNA methylation might be an important mechanism of *BIRC5* dysregulation in breast cancer.

Then, we analyzed the relevance of *BIRC5* expression and different clinicopathological features. In the current work, estrogen receptor (ER) and progesterone receptor (PR) were negatively correlated with *BIRC5* expression. We also confirmed that high *BIRC5* level was associated with increased SBR and NPI, HER-2 positivity, basal-like status, and triple-negative status. Since patients with ER or PR negative, HER-2 positive, basal-like or triple-negative status generally display therapy insensitivity and inferior prognosis, our results suggested that lower expression of *BIRC5* may predict a satisfied clinical outcome of breast cancer.

Next, we checked the prognostic significance of *BIRC5* in breast cancer and demonstrated that high *BIRC5* expression was responsible for shorter relapse-free survival, worse overall survival, reduced distant metastasis-free survival, and increased risk of metastatic relapse event. This was based on survival curves obtained from the Kaplan–Meier Plotter and forest plot generated from the bc-GenExMiner database. Thus, our findings provided evidence that *BIRC5* could be a useful predictive prognostic marker for breast cancer. Indeed, several studies have reported *BIRC5* as a prognostic marker in breast cancer through experimental evidences [25–27]. However, the sample size is small and the evidence is insufficient for a single experimental paper. In the present work, we use several bioinformatics analysis tools, and the combination of bioinformatics analysis tools could provide more sufficient evidence for survival marker validation.

Provided the high *BIRC5* expression was involved in therapy response and worse prognosis, how to handle *BIRC5* overexpressed patients remains a lot of confusion. Here, we showed that a number of commonly used drugs were able to modulate *BIRC5.* For example, cisplatin, epirubicin, doxorubicin, tamoxifen, and lapatinib could decrease *BIRC5* level, while docetaxel could increase *BIRC5* expression. Hormone receptors were negatively correlated with *BIRC5* level as described in Figure 4, and endocrine drug tamoxifen could reduce *BIRC5* level as shown in Figure 6. Therefore, it is interesting to see whether hormone receptor positive patients with BIRC5 overexpression would benefit from *BIRC5* repression. We are currently enrolling patients (half cases with high *BIRC5* expression and half with low expression) to evaluate the effect of *BIRC5*. Remarkably, *BIRC5* has been found to confer resistance to chemotherapy and radiation. Targeting *BIRC5* including antisense oligonucleotide, ribozyme, RNA interference, and cancer vaccine in experimental models improved survival [11,12]. Of *BIRC5*-related signaling pathways, ‘hippo signaling pathway’ and ‘platinum drug resistance’ have been previously confirmed to be responsible for drug resistance [28]. Further investigations are needed to more precisely elucidate the relevance of these signaling pathways.

As a key component of the spindle assembly checkpoint, *CDC20* has been reported in various malignant tumors and exhibits an important role in carcinogenesis and progression [24,29]. High level of *CDC20* was found to be related to aggressive course of breast cancer and poor patient outcome, after 20 years follow-up of 445 patients [30]. After co-expression and correlation analysis using the Oncomine, bc-GenExMiner, and UCSC Xena web-based tools in the present study, we confirmed that *CDC20* gene was positively correlated with *BIRC5* expression. As a matter of fact, both *CDC20* and *BIRC5* were found to be co-expressed in lung adenocarcinoma, endometrial cancer, renal cell carcinoma, and thyroid carcinoma [31–34]. Given that cell cycle process is often coordinated with apoptotic proteins to maintain tissue homeostasis, silencing the expressions of cell cycle protein as well as anti-apoptotic proteins simultaneously could potentially lead to cell cycle arrest and reduce the proliferation of breast cancer cells. Recently, the *CDC20* and *BIRC5* small interfering RNAs delivered by additive polyplexes displayed novel therapy efficacy in breast cancer cell [35]. This pioneering research, along with our bioinformatics analysis, would add a piece of evidence to the emerging idea that *BIRC5* might contribute to breast cancer progression and drug resistance associated with *CDC20* expression.

## Conclusion

The present study validated that *BIRC5* was overexpressed in breast cancer patients and was responsible for a worse survival. *BIRC5* might be adopted as a promising predictive biomarker and potential therapeutic target with co-expressed *CDC20* gene. Further large-scale studies are needed to more precisely confirm the prognostic significance of *BIRC5* in breast cancer treatment.

## Data Availability

The datasets analyzed during the current study are available from the corresponding author on reasonable request.

## Abbreviations

BIRC5: baculoviral inhibitor of apoptosis repeat containing 5
CDC20: *cell-division cycle protein 20*
CI: confidence interval
ER: estrogen receptor
HER-2: human epidermal growth factor receptor-2
HR: hazard ratio
NPI: Nottingham Prognostic Index
PR: progesterone receptor
SBR: Scarff–Bloom–Richardson
TCGA: The Cancer Genome Atlas
